# Evaluation of the effect of nano-hydroxyapatite/silica gel bone graft and/or hemodialysate paste on the regeneration and nano-mechanical properties of calvarial bone in gammairradiated albino rats

**DOI:** 10.1007/s10006-025-01413-8

**Published:** 2025-06-04

**Authors:** Noha S. Zein, Sara El Moshy, Mohammed Khashaba, Eman M. F. El-Maghraby, Engy R. Rashed, Dalia M. Abdel-Hamid

**Affiliations:** 1 Health Radiation Research Department, National Center for Radiation Research and Technology (NCRRT), Egyptian Atomic Energy Authority (EAEA), Cairo, Egypt; 2 Oral Biology Department, Faculty of Dentistry, Cairo University, Cairo, Egypt; 3 Oral and Maxillofacial Surgery Department, Faculty of Dentistry, Cairo University, Cairo, Egypt; 4 Drug Radiation Research Department, National Center for Radiation Research and Technology (NCRRT), Egyptian Atomic Energy Authority (EAEA), Cairo, Egypt; 5 Biomaterials Department, Faculty of Dentistry, Cairo University, Cairo, Egypt

**Keywords:** Gamma radiation, Bone regeneration, Bone graft, Hemodialysate, Nano-mechanical properties

## Abstract

**Purpose:**

Evaluating the effect of synthetic nano-hydroxyapatite/silica gel bone substitute and/or hemodialysate on gamma irradiated bone regeneration and bone nano-mechanical properties in rat model.

**Methods:**

Seventy adult male Wistar albino rats (total of 70 defects, 1 defect/rat) were randomly divided into five groups (14 rats/group); 4 irradiated groups; control (Ir-C) without any treatment, NanoBone^®^-treated (Ir-N), Solcoseryl^®^-treated (Ir-S), NanoBone^®^ + Solcoseryl^®^-treated (Ir-NS) groups, and one non-irradiated NanoBone^®^ + Solcoseryl^®^-treated (Nr-NS) group. Each rat’s calvarium was subjected to a single dose of gamma radiation (12 Gy) followed by a single critical-sized defect creation. Defects were then filled with the assigned treatments except the Ir-C group. Rats were euthanized after 4 weeks. Hematoxylin and Eosin, Masson’s Trichrome staining and nano-indentation were performed for histologic and nano-mechanical properties assessment of specimens.

**Results:**

Scattered thin new bone trabeculae with randomly arranged fine collagen fibrils and extravasated blood were evident in Ir-C group, while thicker new bone trabeculae, well-organized collagen fibers and new blood vessels were observed in all treated groups. Ir-C group showed the lowest significant bone area percent, nano-hardness and indentation modulus values, while Nr-NS group possessed the highest significant percent and values. Ir-C group showed the highest significant indentation modulus to nano-hardness ratio followed by Ir-S group, while the lowest ratios were obtained by the rest of the groups with no significance.

**Conclusions:**

The combination of synthetic nano-hydroxyapatite/silica gel bone substitute and hemodialysate enhanced the quality and quantity of regenerated gamma-irradiated rats’ bones.

**Clinical trial number:**

Not applicable.

## Introduction

The prevalence of head and neck cancers has elevated over the past few years. Surgery in conjunction with chemotherapy and/or radiotherapy are the best treatment methods to date. Oral adverse effects due to radiotherapy include xerostomia, radiation caries, loss of taste sensation, and osteoradionecrosis [1]. Nevertheless, radiotherapy produces changes in bone tissues manifested as death of bone remodeling cells (osteoblasts and osteoclasts) and endothelial cells “hypocellularity”, impaired vascularization “hypovascularity” and deficiency in the amount of oxygen reaching the affected tissues “hypoxia”. This in turn alters bone metabolism and remodeling cycle and reduces calcium and phosphate forming the hydroxyapatite mineral which is responsible for mechanical properties of bone [2].

Different types of bone grafts have been suggested to enhance bone regeneration in defected areas subjected to radiotherapy [3]. Autologous bone is the gold standard for bone graft materials as it shows osteoinductive, osteoconductive and osteogenic properties [4]. However, disadvantages of autograft transplantation include limited tissue availability, prolonged operating time, impaired healing and donor site morbidity especially in older/ or osteoporotic patients [5]. Allografts and xenografts on the other hand show osteoconductive and sometimes osteoinductive properties but have the limitations of immune-mediated rejection and risk of disease transmission [6].

Synthetic bone grafts were manufactured to overcome the drawbacks of the other types. They can be fabricated from different materials as polymers, ceramics, metals and composites [5]. Researchers have thoroughly investigated calcium phosphate ceramics, particularly tricalcium phosphate (TCP) and hydroxyapatite (HA) for bone regeneration applications. Their osteoconductivity and biocompatibility are attributed to their resemblance to the main inorganic constituent of human bones [7].

Implementing the concept of nanotechnology in developing biomaterials has led to producing nano-sized synthetic bone substituting materials with outstanding characteristics. Among those is the nano-crystalline HA silica gel bone graft (NanoBone^®^) that mimics the natural bone structure and improves bone regeneration [8]. The nano-crystalline HA is added to the silica gel matrix in a sol-gel process producing a highly porous nano-textured structure. This serves as a basis for the biomaterial’s exceptional biocompatibility and biodegradability [9, 10].

The highly purified protein-free hemodialysate paste (Solcoseryl^®^) has demonstrated promising pharmacological actions as enhancing oxygen utilization, uptake of glucose by cells, stimulating adenosine triphosphate (ATP), collagen synthesis and energy metabolism promoting angiogenesis while reducing cell apoptosis and oxidative stress. This stimulates cell growth and facilitates wound healing. Solcoseryl^®^ also has beneficial regenerative effects in the treatment of radiation-induced damage as well as disturbances of blood circulation [11]. Furthermore, previous studies have reported the efficacy of Solcoseryl^®^ in accelerating bone regeneration in the bone defects of experimental animals and around implants [12–14].

Therefore, the aim was to evaluate the effect of synthetic nano-hydroxyapatite/silica gel bone substitute (NanoBone^®^) and/or hemodialysate (Solcoseryl^®^) on bone regeneration and nano-mechanical properties of calvarial bone in gamma-irradiated albino rats. The null hypothesis of the present study was that there would be insignificant difference in the histomorphometric analysis of regenerated bone (bone area percentage) and the nano-mechanical properties of gamma-irradiated bone grafted with NanoBone^®^ and Solcoseryl^®^ when compared to non-grafted gamma-irradiated bone in rat animal model after four weeks.

## Methods

### Sample size calculation

To determine the optimal sample size for this study, a statistical power analysis approach is used. The goal is to achieve 80% power (0.8) and a 95% confidence level (α = 0.05), ensuring reliable detection of differences between treatment groups. The sample size for each experimental test was 6 defects per group for the histomorphometric analysis and 8 defects per group for the nano-mechanical properties measurement. Effect size (d) measures the expected difference between groups. An effect size of 0.5 is assumed if prior data isn’t available.

Sample Size Formula:

$$\:n={\left(\frac{{Z}_{\alpha\:/2}+\_{Z}_{\beta\:}}{\varDelta\:}\right)}^{2}\cdot\:{\sigma\:}^{2}$$

Where Z_α/2_ and Z_β_ represent critical z-values for confidence and power, Δ is the effect size, and σ^2^ is variance.

### Animals and grouping

The experiment was conducted in compliance with the protocol approved by the Institutional Animal Care and Use Committee (CU-IACUC), Cairo University (CU III F 29 19). Seventy adult male Wistar albino rats (120–150 g) were enrolled in the study. Rats were kept in polypropylene/stainless steel cages with dimensions of 54, 37 and 27 cm and submitted to suitable ventilation, controlled temperature (25 max 28 °C), max humidity of 55% and 12 h of light/dark cycle with pellet diet and drinkable water ad libitum.

The rats were randomly divided into 5 groups (*n* = 14): Ir-C group (irradiated control without any treatment), Ir-N group (irradiated NanoBone^®^-treated), Ir-S group (irradiated Solcoseryl^®^-treated), Ir-NS group (irradiated NanoBone^®^ + Solcoseryl^®^-treated) and Nr-NS group (non-irradiated NanoBone^®^ + Solcoseryl^®^-treated).

### Gamma irradiation

The rats of Ir-C, Ir-N, Ir-S and Ir-NS groups were subjected to a single dose of 12 Gy of gamma irradiation which was localized on the cranial region by shielding rats’ bodies using a cylindrical lead jacket. Irradiation was carried out at the National Center for Radiation Research and Technology (NCRRT, Cairo, Egypt) utilizing the Cobalt-60 irradiation source (GAMMACELL^®^-220, Nordion International Inc., Kanata, Ontario, Canada) at dose rate of 1.83 KGy/h at the time of the study [15, 16].

### Standardization of the study materials amount

A sterile custom-made circular teflon mold having dimensions of 5 × 1 mm was constructed to simulate the dimensions of the planned surgical bony defect and to standardize the amount of the materials used in the current study. NanoBone^®^ granules were filled into the mold, weighed using an electronic balance with a readability of 0.1 mg (Precise Instrument ltd, Switzerland) and sealed in a sterile container. This process was repeated to obtain a total of 14 sterile containers filled with equal amounts of NanoBone^®^ granules to fill the surgical bony defects of the Ir-N group. For the hemodialysate paste (Solcoseryl^®^), the mold was filled with Solcoseryl^®^ using a sterile syringe weighed before and after dispensing the paste to attain 14 equally filled syringes to be used in the surgical bony defects of the Ir-S group. Lastly, the NanoBone^®^ and Solcoseryl^®^ were mixed in a ratio of 1:1 by weight on a sterile glass slab, placed in sealed containers to fill 28 surgical bony defects; 14 for the Ir-NS group and 14 for the Nr-NS group [13].

### Surgical procedures

**Fig. 1:**
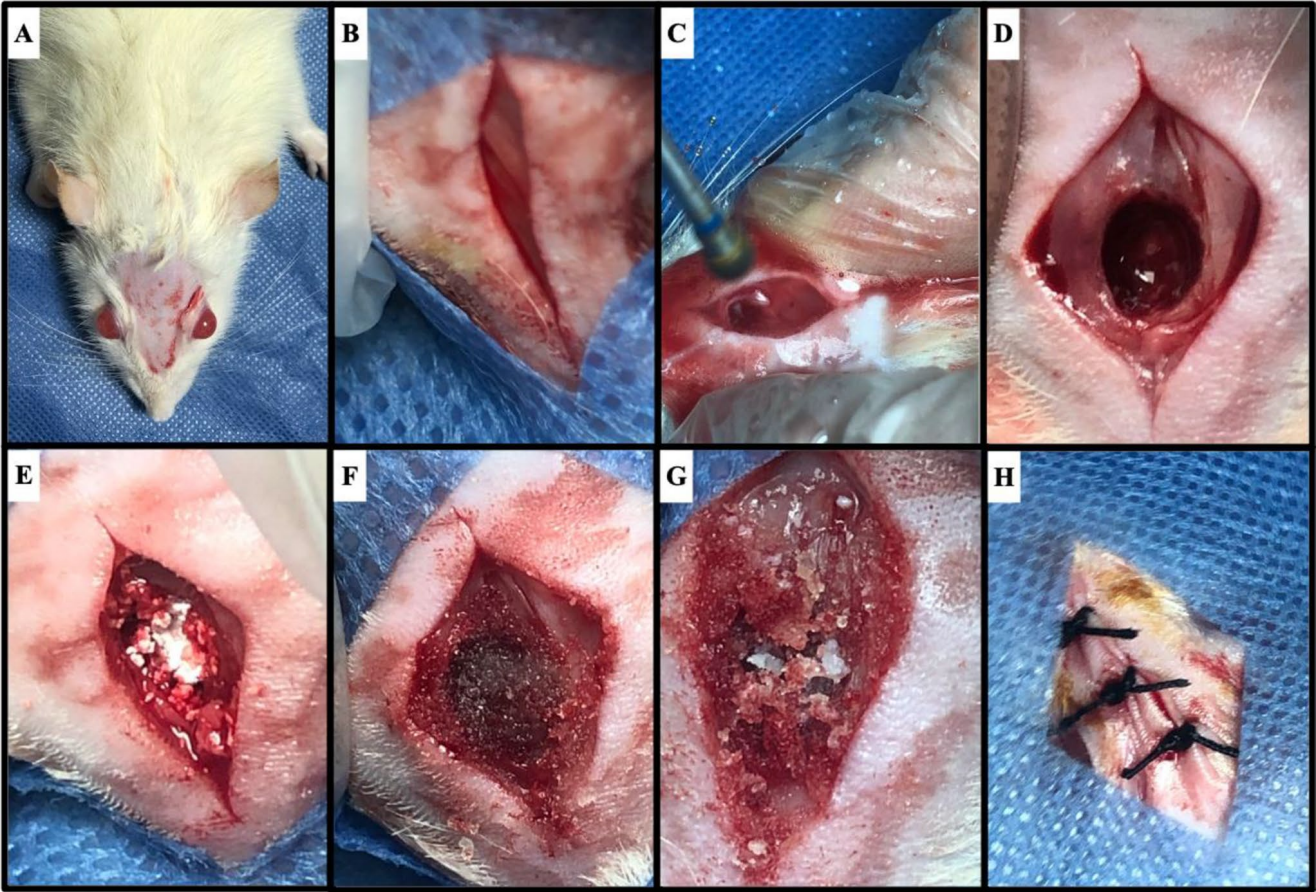
Surgical procedure (**A**) disinfection and shaving of the surgical site (**B**) midline incision (**C**) drilling in the calvarial bone (**D**) critical-sized defect creation (**E**) filling defect with NanoBone^®^ (**F**) filling defect with Solcoseryl^®^ (**G**) filling defect with mixture of NanoBone^®^ + Solcoseryl^®^ (**H**) suturing of soft tissue flaps

Three days following gamma irradiation, surgical procedures were performed on rats’ crania. After anesthetizing each rat using sodium pentobarbital (50 mg/kg bw) [17] and under sterile surgical conditions, midline skin incisions were made followed by elevation of the underlying periosteum. Single centralized critical-sized defect with a 5 mm diameter was performed in the calvarial bone of each rat [18]. Based on previous studies, calvarial bone thickness was found to be 1 mm [19, 20]. The defect’s preparation started using a customized surgical template with the required diameter of the defect (5 mm). This was followed by using 0.5 then 1 mm round burs to drill three holes till the required depth (1 mm) by the defect outline. The depth was confirmed using a graduated periodontal probe. Finally, 5 mm round bur was used to remove the remaining bone guided by the previously drilled holes. The drilling was carried out using low-speed motor with copious irrigation to minimize heat generation and damage to tissues [21]. Extra care was taken to avoid damaging brain and meningeal tissues [18, 22]. Each defect was treated with the pre-weighed study materials according to their group except Ir-C group that was left empty for natural bone healing and finally soft tissue flaps were sutured using interrupted 3/0 black silk sutures (Fig. 1). Analgesic and antibiotic were administered for 7 days to minimize postoperative pain and infection [13, 23]. Rats were euthanized after four weeks by overdose anesthesia [17].

### Histological examination

Thirty calvarial specimens (6 specimens per group) were fixed in 10% formaldehyde solution for 24 h, immersed in 10 EDTA for one month until decalcified, dehydrated using alcohol and xylol and inserted in paraffin to obtain blocks from which 5 μm thickness histological specimens were cut and stained with Hematoxylin & Eosin (H&E). Slides were examined by light microscopy for histological evaluation (Orig. Mag. x100 and x400). Additionally, the specimens were stained with Masson’s trichrome staining for analysis of collagen maturation and examined by the same light microscope (Orig. Mag. x400) [24].

### Histomorphometric analysis

The area percent of newly formed bone was analyzed using an image analysis software (Fiji ImageJ) with Trainable Weka Segmentation and BoneJ plugins in images taken from the H&E-stained sections. This was performed on images obtained with x100 magnification from the center and periphery of the defects. Five fields were measured and averaged from each specimen to obtain mean values of the area percent of newly formed bone [25].

### Nano-mechanical properties measurement

Fourty calvarial specimens (8 specimens per group) were sectioned into rectangular-shaped specimens, embedded in acrylic resin blocks, polished and transferred to the nano-indenter instrument (Nano Indenter^®^ G200, Agilent Technologies, Inc., California, USA) at the National Center for Radiation Research and Technology NCRRT for nano-mechanical properties measurement (nano-hardness and indentation modulus).

Each test specimen was subjected to 9 indents with 25 μm spacing between each indent. A loading profile was created with a maximum load of 3 mN. Collection and analysis of data were carried out using the previously described method by Oliver and Pharr [26]. The depth of penetration and area of indent were automatically recorded and the values were plotted on a graph creating a load displacement-curve for each indent [27]. The nano-hardness (NH) in GPa was calculated using the following equation:

$$\text{NH}=\frac{{P}_{max}}{A}$$

[26].

where *P*_*max*_ is the maximum load applied at the test surface and *A* is the projected contact area at that load.

The elastic modulus (*E*) in GPa of the tested specimen was calculated from the reduced modulus (*E*_*r*_), that is given by the following equation:

$$\text{E}_r=\frac{\sqrt{\pi\:}}{2}\:\frac{S}{\sqrt{A}}$$

[26].

where *S* is the contact stiffness determined by the slope of the initial portion of the unloading curve. The elastic modulus (*E*) of the test specimen was calculated from the equation and described as the indentation modulus (IE):

$$\:\frac{1}{{E}_{r}}=\frac{(1-{v}^{2})}{E}+\frac{(1-{v}_{i}^{2})}{{E}_{i}}$$

[26].

where *E*_*r*_, *E* and *ν* are the reduced modulus, elastic modulus and Poisson’s ratio of the test specimen respectively and *E*_*i*_ and *ν*_*i*_ are the elastic modulus and Poisson’s ratio of the diamond indenter respectively. For the diamond indenter, the elastic constants *E*_*i*_ = 1141 GPa and *ν*_*i*_ = 0.07 were used. Poisson’s ratio for calvarial bone was assumed to be 0.3.

The nano-hardness (NH) and indentation modulus (IE) of the 9 indents/ test specimen were calculated and averaged to obtain mean values for each test specimen. Values were recorded in GPa.

### Statistical analysis

Data analysis was performed using Statistical Package for Social Sciences version 20 (SPSS 20^®^), Graph Pad Prism^®^ and Microsoft Excel 2016. All quantitative data were explored for normality using Shapiro-Wilk and Kolmogorov-Smirnov tests and presented as means and standard deviation values. One-way Analysis of Variance (ANOVA) test was used to compare between different groups followed by Tukey’s Post Hoc test for multiple comparisons.

## Results

### Histological evaluation

**Fig. 2:**
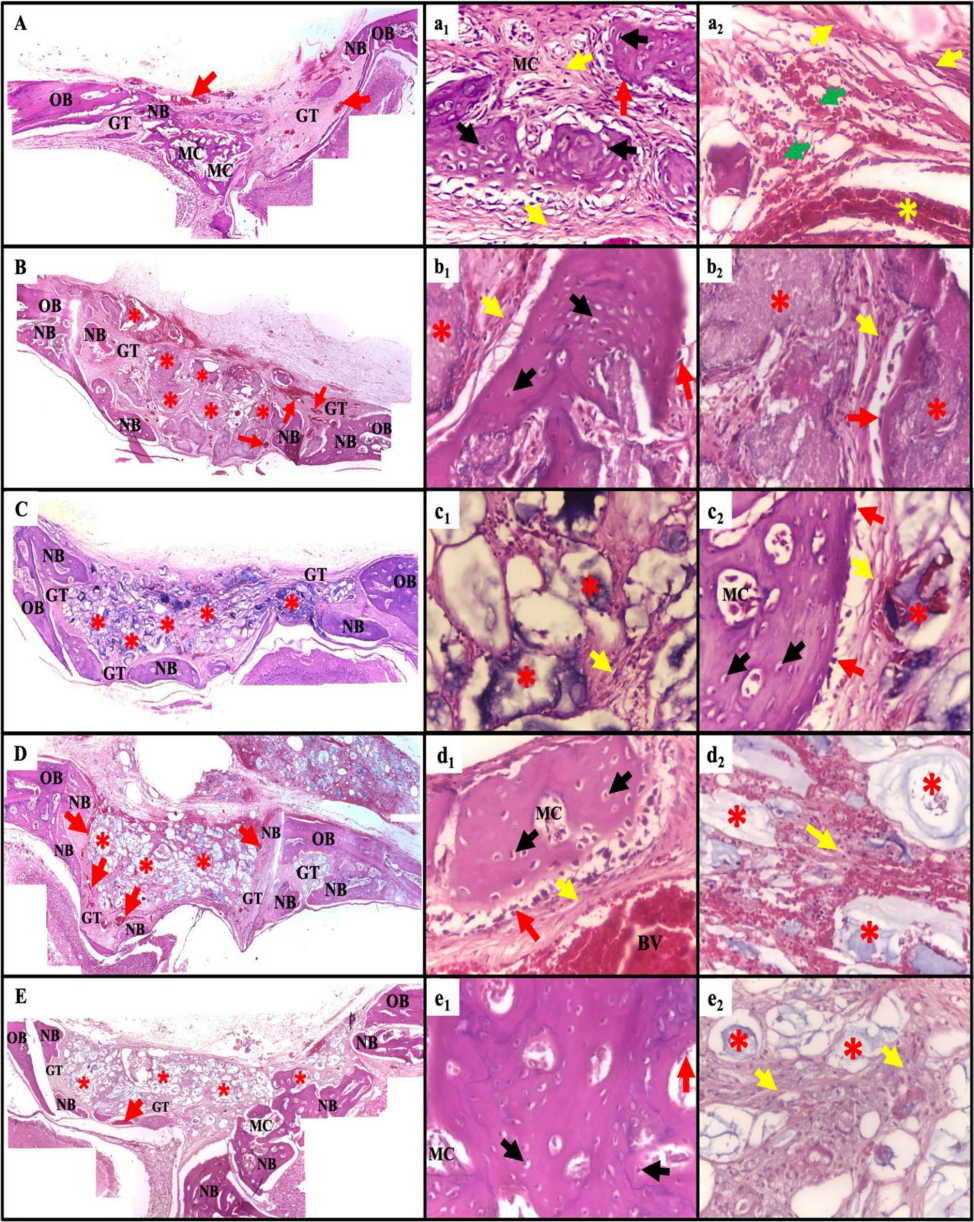
Photomicrographs of the different experimental groups (**A**) Ir-C group (**B**) Ir-N group (**C**) Ir-S group (**D**) Ir-NS group (**E**) Nr-NS group showing the bone defects enclosing granulation tissue (GT), congested blood vessels (red arrows), newly formed bone trabeculae (NB) at the edges of the defect extending from the old bone peripheries (OB) and enclosing marrow cavities (MC) (H&E, Orig. mag. x100). (a_1_-e_2_) are higher magnifications of the different experimental groups representing the newly formed bone with randomly arranged osteocytes (black arrows), osteoblasts (red arrows) lining bone trabeculae, different size marrow cavities (MC) enclosing collagen fibers (yellow arrows) with spindle shape fibroblasts, blood vessels (yellow asterisk) and some extravasated red blood cells (RBCs) (green arrows) (H&E, Orig. Mag. x400)

Examination of the H&E-stained specimens revealed that the bone defects of the irradiated control (Ir-C) group without any treatment were filled with granulation tissue. Few thin newly formed bone trabeculae were observed extending from the defect’s peripheries. These new bone trabeculae were scattered in the granulation tissue filling the defect (Fig. 2A). In the irradiated NanoBone^®^-treated (Ir-N) group, as well as the irradiated Solcoseryl^®^-treated (Ir-S) group, the core of the defect, was mainly filled with remnants of the NanoBone^®^ and Solcoseryl^®^ respectively, with newly formed bone trabeculae from the peripheries and bottom of the bone defect as presented in Fig. (2B) & (2 C) respectively. Meanwhile, the irradiated NanoBone^®^ + Solcoseryl^®^-treated (Ir-NS) group (NS) and the non-irradiated NanoBone^®^ + Solcoseryl^®^-treated (Nr-NS) group revealed thicker newly formed bone trabeculae extending from the defect’s peripheries toward the bottom of the defect as shown in Fig. (2D) & (2E) respectively. Despite the fact that the amount of the newly formed bone trabeculae was greater in the Nr-NS group compared to the Ir-NS group, in both groups the defect core was filled with remnants of the tested materials.

Upon magnification, the newly formed bone trabeculae in all groups enclosed numerous and randomly arranged large-sized osteocytes entrapped in their lacunae. Osteoblasts were found lining the newly formed bone trabeculae (Fig. 2a_1_, b_1_&b_2_, c_2_, d_1_ & e_1_).

Granulation tissue revealed collagen fibrils. The fine collagen fibrils showed random arrangements in some defect areas in the Ir-C group (Fig. 2a_1_&a_2_). On the other hand, they showed increased thickness and some well-organized pattern in the other groups. In addition, well-arranged fine collagen fibrils were spotted at areas of new bone formation surrounding newly formed bone trabeculae and between them (Fig. 2b_1_&b_2_, c_1_&c_2_, d_1_&d_2_ & e_2_).

Regarding the blood supply at the defect sites, there was extravasated blood in the Ir-C group (Fig. 2a_2_) and newly formed blood vessels were detected in all groups especially the Ir-NS group (Fig. 2A, B, C, D and d_1_ & 2E).

**Fig. 3:**
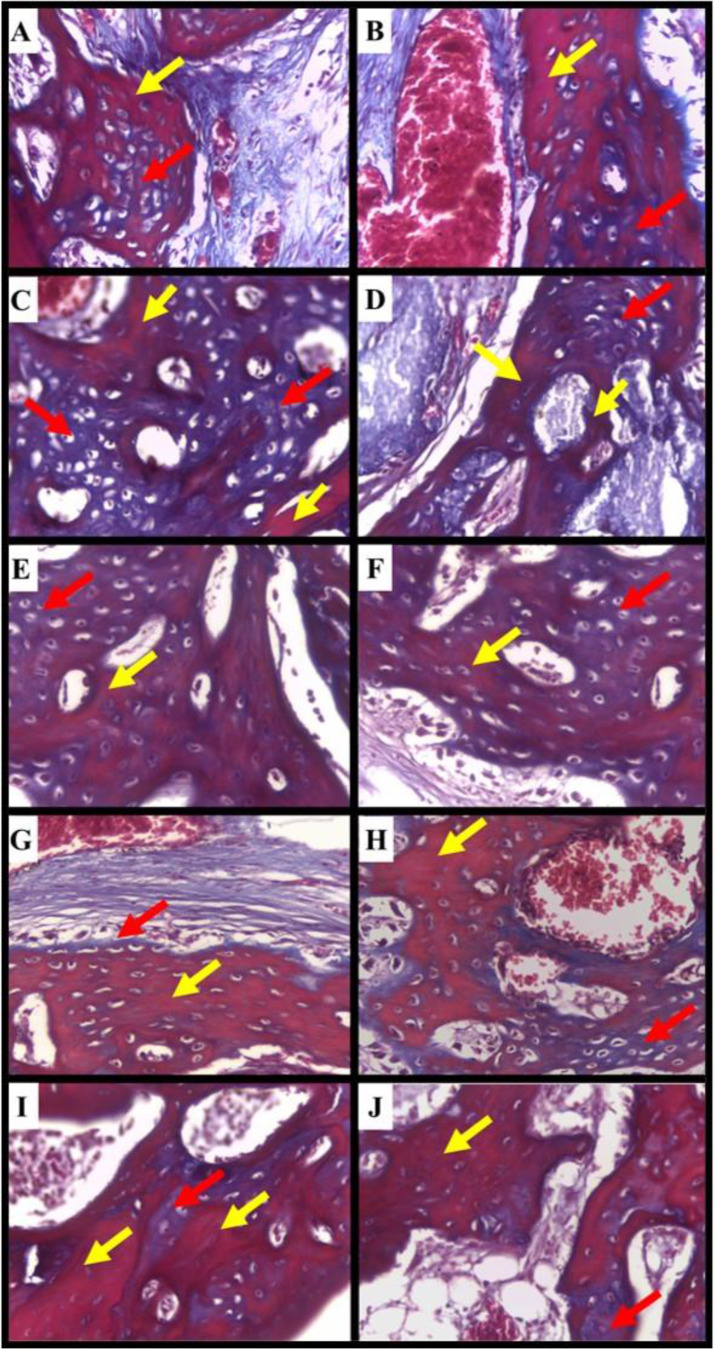
Photomicrographs of the calvarial bone defects (**A**&**B**) the Ir-C group, (**C**&**D**) the Ir-N group, (**E**&**F**) the Ir-S group, (**G**&**H**) the Ir-NS group, (**I**&**J**) the Nr-NS group showing collagen fibers of the newly formed woven bone indicated by the blue color (red arrows) and areas of lamellar bone represented by the red color (yellow arrows) (Masson’s Trichrome, Orig. Mag. x400)

The Masson’s trichrome-stained sections exhibited different levels of collagen maturation. In all groups, the newly formed bone trabeculae exhibited the presence of newly synthesized collagen fibers, which were displayed as a blue color. This indicated the formation of immature bone, with bone areas appearing as red color reflecting the maturation of collagen fibers over time. The amount of mature bone was evident in all experimental groups with an increased amount in the Ir-NS and Nr-NS groups. On the other hand, the areas of immature bone in the Ir-C, Ir-N, and Ir-S groups were clearly demonstrated in the newly formed bone trabeculae (Fig. 3).

### Histomorphometric analysis

**Fig. 4:**
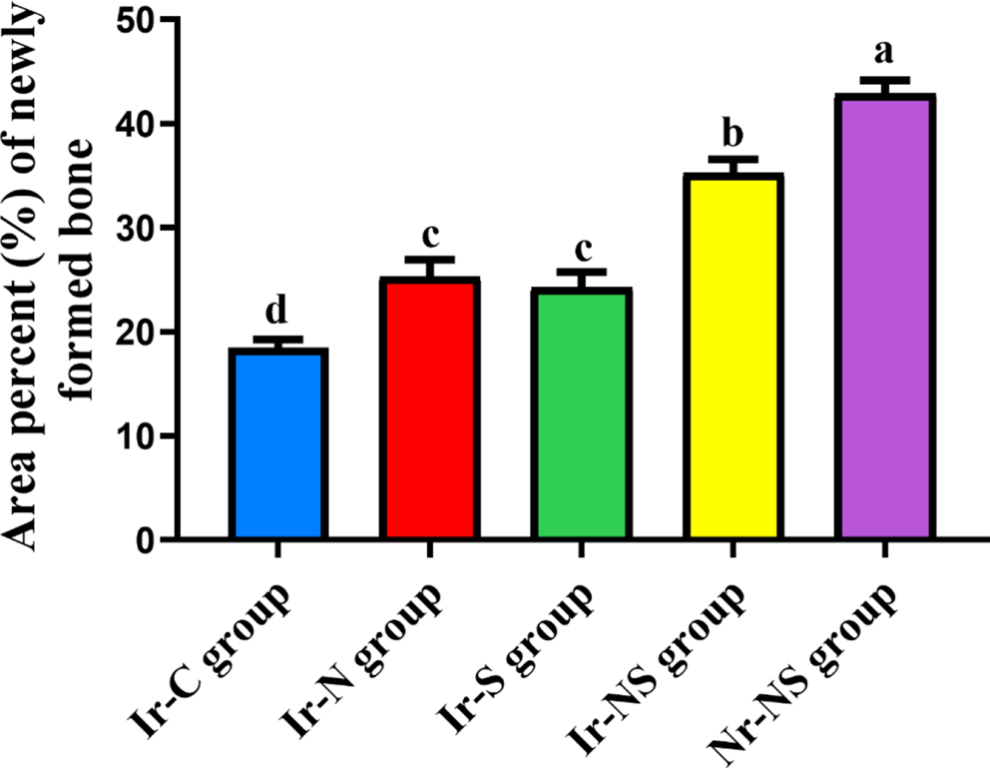
Bar chart showing mean area percent of new bone in the different study groups

The histomorphometric analysis revealed that the Ir-C group showed the lowest significant mean area percent of new bone, followed by the Ir-S group and the Ir-N group with insignificant difference between both groups, followed by the irradiated Ir-NS group and finally the Nr-NS group having the highest significant mean area percent (Table 1; Fig. 4).

**Table 1:** Area percent of new bone in the different study groups

Group	Mean area percent of new bone	Standard deviation (± SD)	*P*-value
Ir-C (irradiated control without any treatment)	18.49 ^**d**^	1.89	< 0.0001*
Ir-N (irradiated Nanobone^®^-treated)	25.30 ^**c**^	3.94	
Ir-S (irradiated Solcoseryl^®^-treated)	24.28 ^**c**^	3.61	
Ir-NS (irradiated Nanobone^®^ + Solcoseryl^®^-treated)	35.27 ^**b**^	3.18	
Nr-NS (non-irradiated Nanobone^®^ + Solcoseryl^®^-treated)	42.83 ^**a**^	3.26	

Different superscript letters indicate significant difference at *P* ≤ 0.05

Same superscript letters indicate insignificant difference at *P* > 0.05

* Significant at *P* ≤ 0.05

### Nano-mechanical properties measurement

**Fig. 5:**
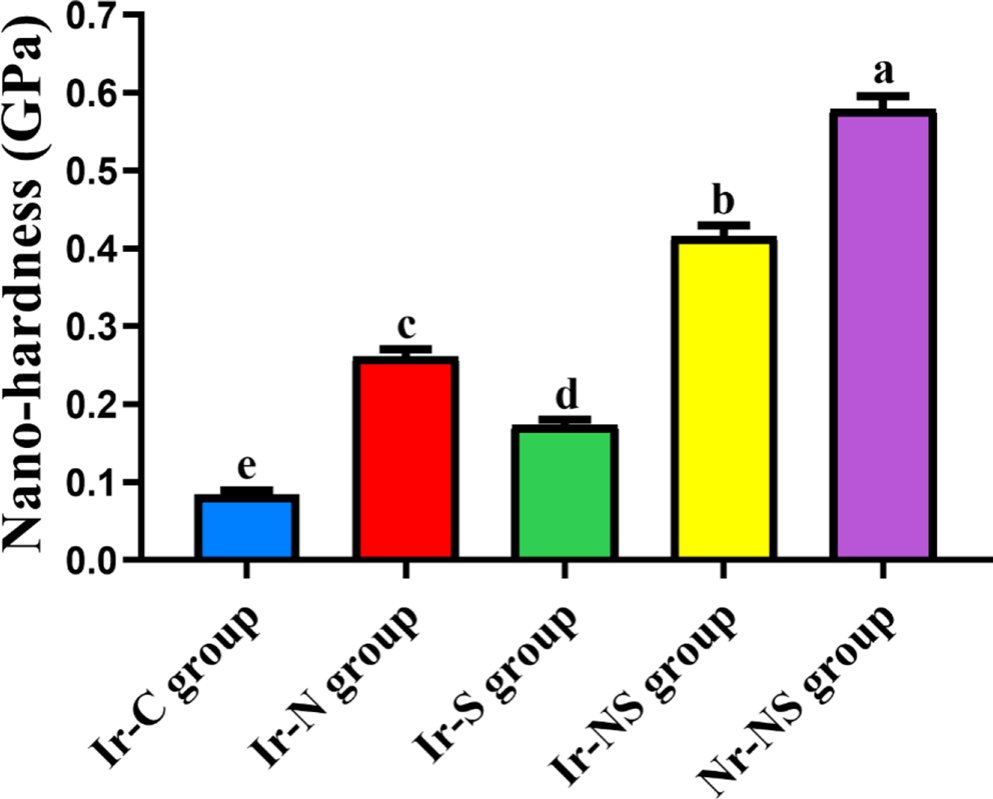
Bar chart showing mean nano-hardness values (GPa) of the different study group

**Fig. 6:**
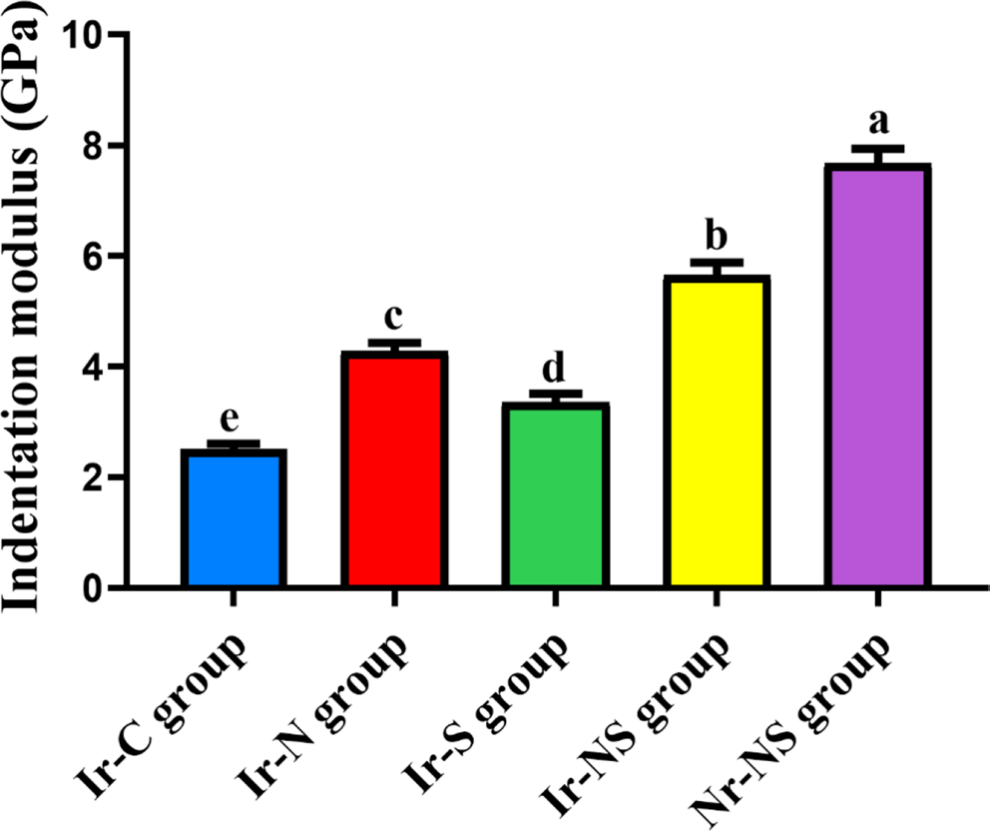
Bar chart showing mean indentation modulus values (GPa) of the different study groups

**Fig. 7:**
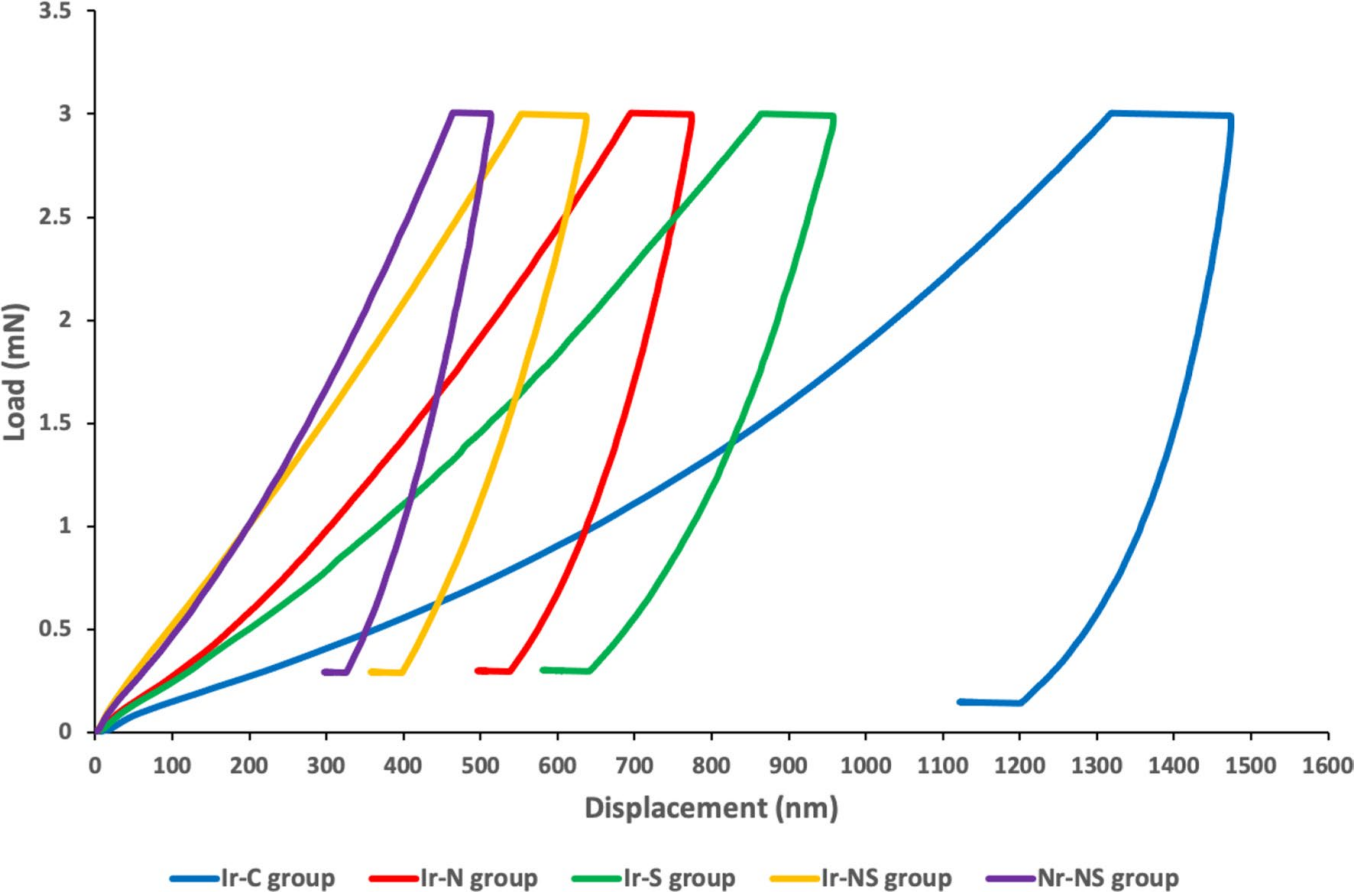
Representative load-displacement curves for the different study groups

The Ir-C group possessed the lowest mean nano-hardness and indentation modulus values, followed by the Ir-S group, followed by Ir-N group, followed by the Ir-NS group and finally the Nr-NS group showed the highest values with significant differences between all groups (Tables 2 and 3; Figs. 5 and 6). Representative load-displacement curves for the different groups are presented in Fig. 7.

**Table 2:** Nano-hardness (GPa) values of the different study groups

Group	Mean nanohardness (GPa)	Standard deviation (± SD)	*P*-value
Ir-C (irradiated control without any treatment)	0.085 ^**e**^	0.013	< 0.0001*
Ir-N (irradiated Nanobone^®^-treated)	0.261 ^**c**^	0.028	
Ir-S (irradiated Solcoseryl^®^-treated)	0.174 ^**d**^	0.020	
Ir-NS (irradiated Nanobone^®^ + Solcoseryl^®^-treated)	0.416 ^**b**^	0.039	
Nr-NS (non-irradiated Nanobone^®^ + Solcoseryl^®^-treated)	0.579 ^**a**^	0.047	

Different superscript letters indicate significant difference at *P* ≤ 0.05

* Significant at *P* ≤ 0.05

**Table 3:** Indentation modulus (GPa) values of the different study groups

Group	Mean indentation modulus (GPa)	Standard deviation (± SD)	*P*-value
Ir-C (irradiated control without any treatment)	2.509 ^**e**^	0.267	< 0.0001*
Ir-N (irradiated Nanobone^®^-treated)	4.282 ^**c**^	0.406	
Ir-S (irradiated Solcoseryl^®^-treated)	3.350 ^**d**^	0.438	
Ir-NS (irradiated Nanobone^®^ + Solcoseryl^®^-treated)	5.649 ^**b**^	0.648	
Nr-NS (non-irradiated Nanobone^®^ + Solcoseryl^®^-treated)	7.671 ^**a**^	0.755	

Different superscript letters indicate significant difference at *P* ≤ 0.05

* Significant at *P* ≤ 0.05

**Fig. 8:**
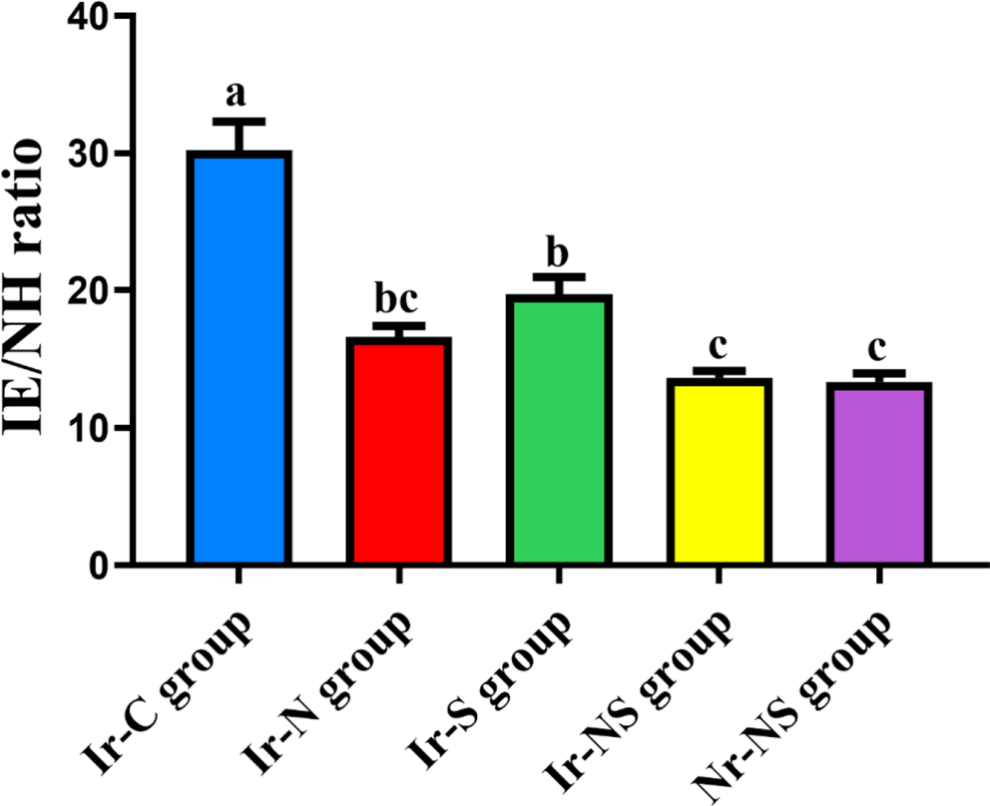
Bar chart showing mean IE/NH ratios of the different study groups

The comparison between the indentation modulus to nano-hardness (IE/NH) ratio revealed that the Ir-C group showed the highest IE/NH ratio, followed by the Ir-S group, while the lowest IE/NH ratios were obtained by the rest of the groups where there were insignificant differences between the Ir-N, the Ir-NS and the Nr-NS groups as presented in Table 4; Fig. 8.

**Table 4:** IE/NH ratios of the different study groups

Group	Mean IE/NH ratio	Standard deviation (± SD)	*P*-value
Ir-C (irradiated control without any treatment)	30.217 ^a^	5.891	< 0.0001*
Ir-N (irradiated Nanobone^®^-treated)	16.601 ^bc^	2.278	
Ir-S (irradiated Solcoseryl^®^-treated)	19.562 ^b^	3.696	
Ir-NS (irradiated Nanobone^®^ + Solcoseryl^®^-treated)	13.629 ^c^	1.445	
Nr-NS (non-irradiated Nanobone^®^ + Solcoseryl^®^-treated)	13.327 ^c^	1.758	

Different superscript letters indicate significant difference at *P* ≤ 0.05

Same superscript letters indicate insignificant difference at *P* > 0.05

* Significant at *P* ≤ 0.05

## Discussion

The rat’s calvarium has been chosen in the present study as it permits the creation of uniform, standardized and reproducible defects that can be easily evaluated [28]. A single dose of 12 Gy was delivered to the cranial region of the rats which is equivalent to a fractionated therapeutic dose of 60 Gy that is administered to patients in the treatment of head and neck cancer [15]. Nano-indentation is a non-destructive testing technique where a controlled load is applied to the surface of the tissue to produce localized surface deformation [29]. Therefore, the nano-indentation testing was the technique of choice for the determination of the nano-mechanical properties of the regenerated gamma-irradiated calvarial bones in the current study.

The histological evaluation of Ir-C group revealed abundant areas of granulation tissue with a few thin new bone trabeculae in addition to fine randomly arranged collagen fibers and congested blood vessels with some extravasated red blood cells. Interestingly, the histomorphometric results of Ir-C group demonstrated the lowest significant mean area % of new bone. These findings may be attributed to the adverse impact of gamma irradiation on bone that were manifested as inflammatory cellular infiltration, reduction in both osteogenic cell number and amount of blood flow reaching bone in addition to elevation of bone resorption rates [30]. Marx and Johnson [31] were the first to believe that hypoxia, hypovascularity, and hypocellularity are the main concerns associated with radiotherapy especially if accompanied by surgical intervention. The current findings are in accordance with those of Bléry et al. [32] who reported presence of fibrosis, fewer blood vessels and cell depletion in irradiated rats’ mandibles. Curi et al. [33] also observed hyperemia, loss of cells and vascular content in irradiated mandibular samples of radiotherapeutic patients. Similarly, Sório et al. [34] reported minimal evidence of new bone formation with disorganized granulation tissue, reduced osteocytes and osteoblasts and decreased vascularization in irradiated rats’ calvarial defects.

The Ir-N group specimens showed remnants of NanoBone^®^ with new formed bone trabeculae together with dense collagen fibers having some well-organized pattern, well-arranged fine collagen fibrils and new blood vessels. Furthermore, the histomorphometric analysis declared significantly higher mean area percent of new bone compared to Ir-C group. The ability of NanoBone^®^ regarding improving quality and quantity of gamma-irradiated bone can be due to the chemical and structural similarity between the nano-hydroxyapatite of NanoBone^®^ and natural bone. Moreover, the silica gel matrix forms a scaffold that has a strong impact on differentiation and activation of bone remodeling cells in addition to collagen synthesis and bone matrix mineralization enhancement [10, 35]. Thus, NanoBone^®^ enhanced the healing power of irradiated bone. Regarding the revascularization potential of NanoBone^®^, it was demonstrated that it was able to form an entirely new network of micro-vessels after only 10 to 15 days in mice [36]. The results of the current study are similar to those of AbdelHamid et al. [13] who confirmed the presence of a network of new bone trabeculae in rabbits only 14 days after the treatment with NanoBone^®^. Furthermore, Wähnert et al. [37] reported significantly higher bone volume with NanoBone^®^ compared to untreated control group 30 days post-operatively. Moreover, Ghanaati et al. [38] have proven the generation of new bone trabeculae and numerous blood vessels after using Nanobone^®^ in the maxillary sinuses of chemo and/or radiotherapeutic patients.

The Ir-S group specimens exhibited Solcoseryl^®^ remnants along with new bone trabeculae, well-organized dense collagen fibers, fine collagen fibrils and new blood vessels. Besides, it exhibited significantly higher mean area percent of new bone compared to the Ir-C group. However, it was insignificantly different than the Ir-N group as detected by the histomorphometric analysis. The favorable results of Solcoseryl^®^ may be attributed to its therapeutic potentials as regards protecting cells against ischemic and hypoxic damage via enhancing the uptake of oxygen and glucose, inhibiting inflammatory and apoptotic processes, reducing oxidative stress [39], accelerating wound healing and tissue remodeling by stimulating migration of cells to damaged tissues, in addition to increasing blood circulation [40]. It has also been reported that Solcoseryl^®^ can modify the cellular damage caused by gamma irradiation by reducing post-irradiation breaks of single-stranded DNA [41]. Solcoseryl^®^ exhibited promising results concerning regeneration of bone as presented by Abdel-Hamid et al. [13] who confirmed that it improved the quantity and quality of newly formed bone in comparison to control. Interestingly, El-Sayyad et al. [14] noticed an elevated cellular activity, progressive healing and higher mean area percent of bone associated with Solcoseryl^®^ compared to control. Also, Fukuda et al. [42] and Ochi et al. [12] have proven the effectiveness of Solcoseryl^®^ in the formation of new bone around dental implants.

Thicker new bone trabeculae were detected in both Ir-NS and Nr-NS groups with remnants of the testing materials compared to all other groups along with well-arranged collagen fibrils and new blood vessels. However, Ir-NS group exhibited significantly higher mean area percent of new bone than the other groups except the Nr-NS group, which showed the highest significant mean area percent of newly formed bone. This may be due to the synergistic effect of the two tested materials together, improving the quality and quantity of regenerated bone. Meanwhile, the higher significance of Nr-NS group compared to Ir-NS may result from the extensive damage caused by gamma radiation, which was not completely overcome [43].

The results of Nr-NS group are consistent with those of Abdel-Hamid et al. [13] and ElSayyad et al. [14] who reported that combining Solcoseryl^®^ and synthetic bone graft brought about the highest significant mean area percent of newly formed bone. Additionally, Hu et al. [44] revealed that non-irradiated regenerative rats’ calvaria demonstrated better bone morphology and osseointegration than irradiated ones. Furthermore, Nussenbaum et al. [43], reported that despite the significant healing of irradiated rats’ calvarial defects treated with gene therapy, six weeks were not enough to eliminate the damage entirely.

Results of nano-mechanical properties revealed that Ir-C group showed the lowest significant mean nano-hardness and indentation modulus. This could be attributed to the deleterious effect of gamma radiation on the structure and composition of bone as it causes crystallization disorders, lowers bone mineral density (BMD), additionally, it reduces collagen synthesis, degrades its molecules and affects its cross-linking by the action of reactive oxygen species (ROS), thus disrupts the interaction of the collagen matrix with the mineral phase [45].

The current findings are in agreement with Limirio et al. [46], Soares et al. [47], Bartlow et al. [48] and Oest et al. [49] who assessed the effect of radiotherapy on animals’ bones. They reported that the mechanical properties of irradiated groups were significantly lower than those of the non-irradiated ones.

Ir-S group revealed significantly higher nano-mechanical properties compared to Ir-C group, followed by Ir-N then Ir-NS group with significant differences between these groups. Nevertheless, Nr-NS group showed the highest significant values among all groups. This may be due to the improvement in bone quality by the tested materials each alone or in combination.

Regarding the effect of Solcoseryl^®^ on the mechanical performance of regenerated bone, Ochi et al. [12] demonstrated it significantly increased the implant removal torque inserted in rabbits’ femurs which could reflect enhancement of mechanical properties of regenerated bone [50].

Studies have shown that deposition of calcium and phosphorus increases in defect sites after implantation of NanoBone^®^ reaching a Ca: P ratio resembling that of normal bone [51]. This increase in the mineral content and ratio is correlated with an increase in BMD which influences the bone mechanical properties [52, 53]. Moreover, it has been suggested that the large surface area and the nanometer-sized pores within NanoBone^®^ granules enhance its mechanical stability [36, 37]. In the current study, the presence of significant amount of collagen fibers, some of which are mature, suggests new bone formation following mineralization of its matrix thus, reflecting the positive effects of different treatments on bone regeneration and hence its mechanical properties [3]. The highest significant nano-mechanical properties of Nr-NS group could be attributed to both materials acting synergistically promoting the quality and mechanical properties of bone.

The IE/NH ratio represents the relation between the elastic and plastic properties of bone (viscoelasticity) and indicates the performance of bone throughout the process of indentation [54]. The comparison between the IE/NH ratios of different groups revealed that Ir-C group demonstrated the highest significant ratio followed by Ir-S group. Meanwhile, the rest of the groups showed the lowest ratios with insignificance in between. These results could be accredited to the difference in degree of mineralization and collagen matrix organization between groups. As mentioned previously, gamma radiation affects the mineralization and collagen synthesis within the bone structure resulting in destruction of bone architecture. This was reflected by the elevation of IE/NH ratio of Ir-C group. Sun et al. [55] revealed reduction in BMD, deterioration of nano-mechanical properties and elevation in IE/NH ratio of rats’ femurs due to reduced degree of mineralization and distorted collagen matrix in defective bones. This may further explain the significantly lower ratios belonging to the grafted groups of our study especially those treated with NanoBone^®^ alone (Ir-N) or in combination with Solcoseryl^®^ (Ir-NS) owing to the positive impact of the NanoBone^®^ chemical and structural properties on bone mineralization and collagen matrix organization.

Based on the results of this study, the null hypothesis was rejected as there was a significant difference in the histomorphometric analysis of the regenerated bone (bone area percent) and the nano-mechanical properties of gamma-irradiated bone; grafted with NanoBone^®^ and Solcoseryl^®^, compared to non-grafted gamma-irradiated bone in rats after four weeks.

## Conclusions

Within limitations of the current investigation, it could be concluded that the combination of NanoBone^®^ bone graft and Solcoseryl^®^ paste could be a promising treatment to promote the regeneration of radiotherapy-compromised bone following surgical procedures that may include dental extraction, replacement of bony defects or tumor excision. The overall results revealed that the combined treatment increased the area percent of newly formed bone and improved its nano-mechanical properties, thus it enhanced the quality and quantity of regenerated gamma-irradiated bone.

## Data Availability

No datasets were generated or analysed during the current study.
